# Multidimensional analyses of the pathomechanism caused by the non-catalytic *GNE* variant, c.620A>T, in patients with GNE myopathy

**DOI:** 10.1038/s41598-022-26419-0

**Published:** 2022-12-16

**Authors:** Wakako Yoshioka, Aritoshi Iida, Kyuto Sonehara, Kazuki Yamamoto, Yasushi Oya, Madoka Mori-Yoshimura, Takashi Kurashige, Mariko Okubo, Megumu Ogawa, Fumihiko Matsuda, Koichiro Higasa, Shinichiro Hayashi, Harumasa Nakamura, Masakazu Sekijima, Yukinori Okada, Satoru Noguchi, Ichizo Nishino

**Affiliations:** 1 Department of Neuromuscular Research, National Institute of Neuroscience, National Center of Neurology and Psychiatry (NCNP), 4-1-1 Ogawa-Higashi, Kodaira, Tokyo 187-8502 Japan; 2 Medical Genome Center, NCNP, Kodaira, Japan; 3 Department of Statistical Genetics, Osaka University Graduate School of Medicine, Suita, Japan; 4 Integrated Frontier Research for Medical Science Division, Institute for Open and Transdisciplinary Research Initiatives (OTRI), Osaka University, Suita, Japan; 5 Department of Computer Science, Tokyo Institute of Technology, Yokohama, Japan; 6 Department of Neurology, National Center Hospital, NCNP, Kodaira, Japan; 7 Department of Neurology, National Hospital Organization Kure Medical Center and Chugoku Cancer Center, Kure, Japan; 8 Center for Genomic Medicine, Kyoto University Graduate School of Medicine, Kyoto, Japan; 9 Department of Genome Analysis, Institute of Biomedical Science, Kansai Medical University, Hirakata, Japan; 10 Department of Clinical Research Support, Clinical Research & Education Promotion Division, National Center Hospital, NCNP, Kodaira, Japan

**Keywords:** Genetics, Neurology

## Abstract

GNE myopathy is a distal myopathy caused by biallelic variants in *GNE*, which encodes a protein involved in sialic acid biosynthesis. Compound heterozygosity of the second most frequent variant among Japanese GNE myopathy patients, *GNE* c.620A>T encoding p.D207V, occurs in the expected number of patients; however, homozygotes for this variant are rare; three patients identified while 238 homozygotes are estimated to exist in Japan. The aim of this study was to elucidate the pathomechanism caused by c.620A>T. Identity-by-descent mapping indicated two distinct c.620A>T haplotypes, which were not correlated with age onset or development of myopathy. Patients homozygous for c.620A>T had mildly decreased sialylation, and no additional pathogenic variants in *GNE* or abnormalities in transcript structure or expression of other genes related to sialic acid biosynthesis in skeletal muscle. Structural modeling of full-length GNE dimers revealed that the variant amino acid localized close to the monomer interface, but far from catalytic sites, suggesting functions in enzymatic product transfer between the epimerase and kinase domains on GNE oligomerization. In conclusion, homozygotes for c.620A>T rarely develop myopathy, while symptoms occur in compound heterozygotes, probably because of mildly decreased sialylation, due to partial defects in oligomerization and product trafficking by the mutated GNE protein.

## Introduction

GNE myopathy (previously known as distal myopathy with rimmed vacuoles, hereditary inclusion body myopathy, or Nonaka myopathy) is a rare autosomal recessive disease, the symptoms of which typically first occur in around the third decade of life. Distal muscles are preferentially affected, and the condition develops to involve other muscles, leading to loss of ambulation^1^. GNE myopathy is caused by biallelic variants in the *GNE* gene, which encodes a bifunctional enzyme, UDP-N-acetylglucosamine 2-epimerase/N-acetylmannosamine kinase (GNE/MNK kinase), that catalyzes critical steps in sialic acid (SA) biosynthesis^1^. The underlying cause of muscle weakness in patients with GNE myopathy is considered to be hyposialylation in muscle tissues because GNE variants lead to decreased SA production^2,3^ and oral SA supplementation prevented and arrested muscle phenotypes in mouse models^4,5^. Clinical trials in human are currently underway^6,7^.

There are two potential founder variants in Japanese patients with GNE myopathy, c.620A>T and c.1807G>C, which are missense variants encoding p.D207V and p.V603L amino acid changes, respectively^8^. The allele frequencies of the second most common variant (c.620A>T) are approximately 22.4% and 25.5% in Japanese and Chinese patients, respectively, with GNE myopathy; however, only three and two homozygous patients have been identified in each respective country^8,9^. Further, c.620A>T is associated with milder disease symptoms^8–10^, and an asymptomatic homozygous individual was identified^8,11^, who is currently around 80 years old, with no muscle symptoms.

Given the relatively small number of patients with GNE myopathy homozygous for c.620A>T, their milder phenotypes, and the report of an asymptomatic older homozygote, we hypothesized that homozygosity for c.620A>T is insufficient for myopathy development, and that the muscle weakness in homozygous patients can be attributed to additional genetic alterations around *GNE* or other factors that modify the SA biosynthesis pathway. Here, we screened for additional genetic variation around *GNE* in homozygous patients using genome sequence data from patients, and explored the origin of c.620A>T-alleles in patients homozygous for the variant and the healthy homozygote, using an identity-by-descent (IBD) mapping approach. We also conducted haplotype analysis of patients with compound heterozygous *GNE* variants and healthy individuals in the general population who carry the variant. Further, we screened for genetic alterations and analyzed the expression levels of other genes involved in SA biosynthesis. In addition, we applied three-dimensional structural modeling to characterize the effects of c.620A>T on GNE protein oligomers.

## Results

### Low frequency of patients with GNE myopathy homozygous for c.620A>T based on the estimated number of homozygotes in the Japanese general population

From the Nagahama cohort study, the *GNE* c.620A>T allele frequency in the Japanese general population was calculated as 0.00161 (22/13,686 alleles), while that of c.1807G>C was 0.00089 (12/13,686 alleles). Based on these allele frequencies and the national population of Japan > 28 years old (the average onset age of GNE myopathy in Japanese patients), the expected numbers of c.620A>T and c.1807G>C homozygotes were estimated to be 238 and 73, respectively. In contrast, the numbers of identified patients were 3 and 92, respectively, hence the detection rates of homozygosity in patients were estimated as 0.013 for c.620A>T and 1.260 for c.1807G>C. Similarly, the rates of these two variants among 730 alleles detected in 365 Japanese patients with GNE myopathy were 43.6% (318/730 alleles) for c.1807G>C and 25.3% (185/730 alleles) for c.620A>T, and homozygous rates by allele were 57.9% (184/318 alleles) and 3.2% (6/185 alleles) for c.1807G>C and c.620A>T, respectively. Notably, we also identified one healthy older individual (H1) homozygous for c.620A>T. There were 179 and 134 compound heterozygous c.620A>T and c.1807G>C alleles, respectively.

### Identification of two c.620A>T founder haplotypes

**Figure 1:**
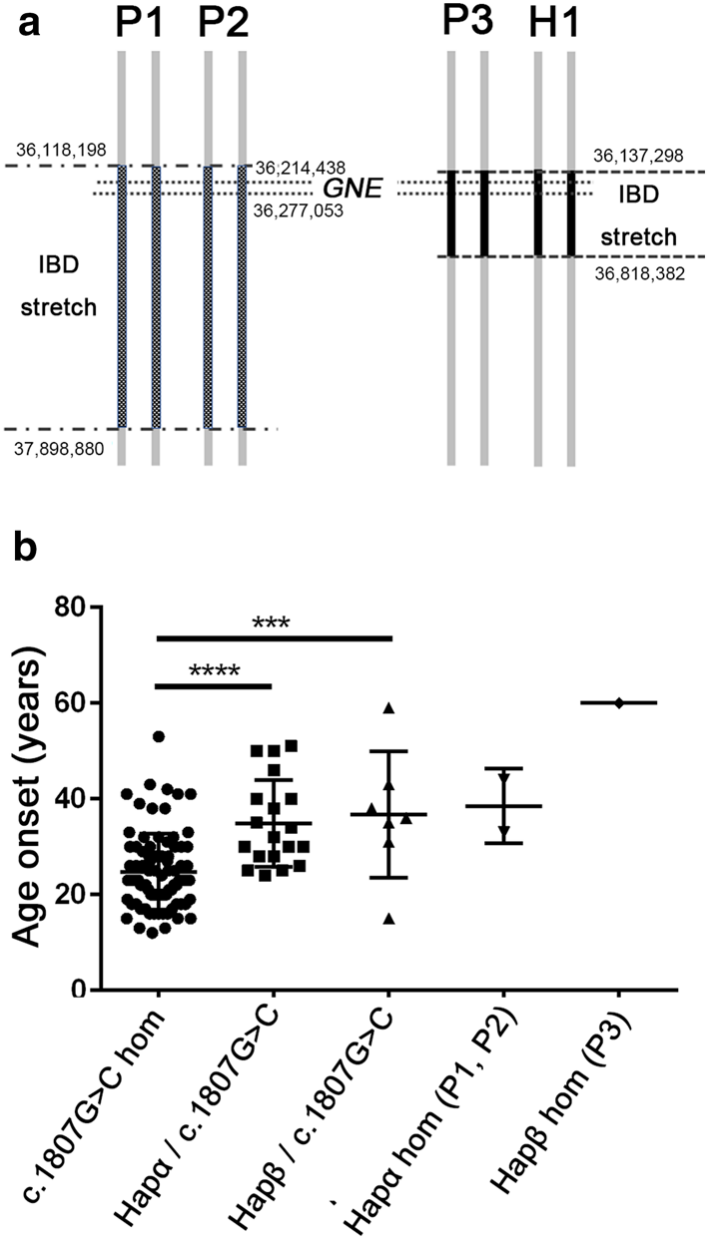
Two distinct haplotypes of patients carrying c.620A>T. (**a**) Two distinct haplotypes carrying the c.620A>T allele. IBD analysis of c.620A>T homozygotes. An IBD stretch spanning 1780 kb (Chr9: 36,118,198–37,898,880), including the whole of *GNE*, was identified in P1 and P2. A different IBD stretch, spanning 680 kb (Chr9: 36,137,298–36,818,382), and also including the whole of *GNE*, was identified in P3 and H1. (**b**) Age at onset of patients homozygous for c.1807G>C and c.620A>T. Haplotype α was shared by P1 and P2. Haplotype β was shared by P3 and H1. Age at onset of c.620A>T/c.1807G>C compound heterozygous patients (hapα: n = 19, hapβ: n = 7) was later than in those homozygous for c.1807G>C (n = 84). There was no significant difference between patients carrying Haplotype α and Haplotype β. The ages at onset of P1, P2, and P3 were 37, 34, and 60 years, respectively. *p < 0.05, **p < 0.01, ***p < 0.001, ****p < 0.0001.

To characterize whether c.620A>T has a common ancestor, we performed IBD analysis of four individuals homozygous for this variant (three patients (P1–P3) and one healthy individual (H1)). An IBD stretch spanning 1780 kb (Chr9: 36,118,198–37,898,880), including the whole *GNE* region, was shared by P1 and P2, and another IBD stretch which spanned 680 kb (Chr9: 36,137,298–36,818,382), also including the whole of *GNE*, was shared by P3 and H1 (Fig. 1a), indicating the presence of two ancestors with different haplotypes (Haplotypes α and β) with the c.620A>T allele. There was no correlation between haplotype and disease development in homozygotes.

The two haplotypes could be distinguished by typing four common variants; variants in [34,214,971, 36,216,426, 36,217,798, and 36,220,134] of chromosome 9 are [G, G, G, C] for Haplotype α and [A, A, A, T] for Haplotype β, respectively (Table1). We also determined the haplotypes carrying the c.620A>T allele in heterozygous individuals, to determine the distribution of each haplotype in compound heterozygous patients with GNE myopathy and the Japanese general population. We found that 73.1% (19/26) of c.620A>T/c.1807G>C compound heterozygous patients and 77.8% (7/9) of c.620A>T heterozygous general individuals had Haplotype α, while 26.9% (7/26) of c.620A>T/c.1807G>C compound heterozygous patients and 22.2% (2/9) of individuals from the general population heterozygous for c.620A>T had Haplotype β (Table 1).

**Table 1:** Two haplotypes harboring *GNE* c.620A>T.

		Age at onset (years)	Common variant haplotypes								Risk locus			Haplotype set
			Position in chromosome 9											
			36,214,971		36,216,426		36,217,798		36,220,134			36,246,117		
c.620A>T homozygotes	P1	Late 30 s	G	G	G	G	G	G	**C**	**C**		**A**	**A**	**α/α**
	P2	Mid-30 s	G	G	G	G	G	G	**C**	**C**		**A**	**A**	**α/α**
	P3	Early 60 s	**A**	**A**	**A**	**A**	**A**	**A**	T	T		**A**	**A**	**β/β**
	H1	Healthy	**A**	**A**	**A**	**A**	**A**	**A**	T	T		**A**	**A**	**β/β**
c.620A>T/c.1807G>C compound heterozygous patients	P4–22	34.8 ± 8.8	G	G	G	G	G	G	**C**	T		**A**	T	**α/–**
	P23–29	36.7 ± 12.2	**A**	G	**A**	G	**A**	G	T	T		**A**	T	**β/–**
c.620A>T heterozygous healthy individuals	U1–7	Healthy	G	G	G	G	G	G	**C**	T		**A**	T	**α/–**
	U8–9	Healthy	**A**	G	**A**	G	**A**	G	T	T		**A**	T	**β/–**

Bases altered from the reference sequence are underlined and in bold. P1–3: three patients homozygous for c.620A>T. H1: healthy individual homozygous for c.620A>T. P4–13: ten patients with compound heterozygous c.620A>T/c.1807G>C alleles. U1–9: individuals carrying c.620A>T from public data (U1–6 from the Biobank Japan Database, U7–9 from the Nagahama cohort study).

### Patients with c.620A>T on the two haplotypes shared similar age of disease onset

We analyzed whether patients with different haplotypes showed varying ages of disease onset; however, there was no difference in age at onset between compound heterozygous patients with c.620A>T on Haplotype α/c.1807G>C (n = 19) and those with c.620A>T on Haplotype β/c.1807G>C (n = 7), while both had later disease onset compared to patients homozygous for c.1807G>C (n = 84) (Fig. 1b).

### No additional variants or abnormal expression of genes associated with SA metabolism in *GNE* c.620A>T homozygotes

**Figure 2:**
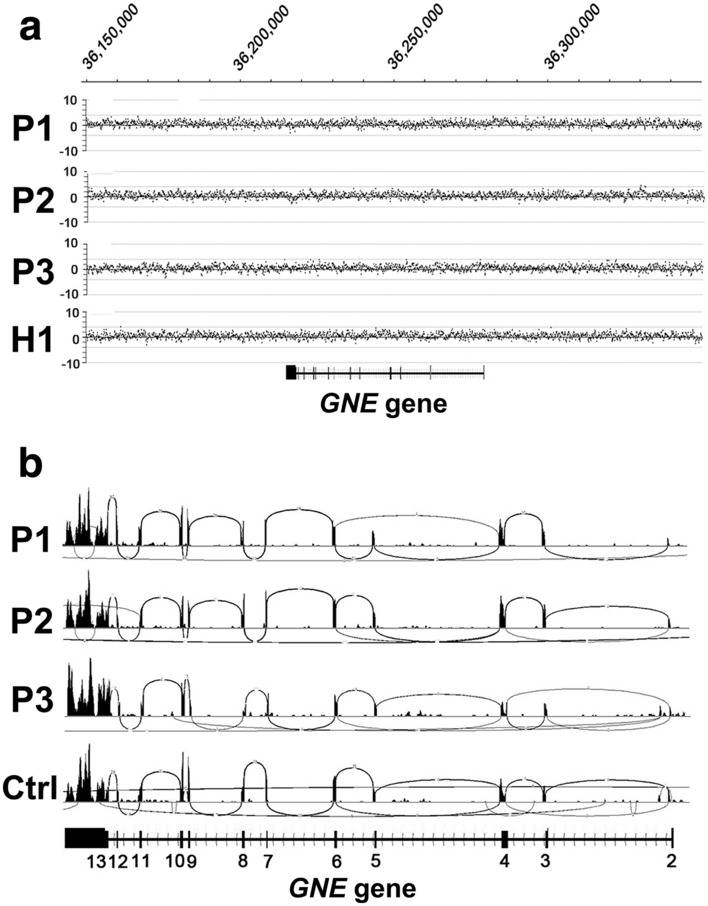
CNV analysis of *GNE* and structure of the *GNE* transcript in skeletal muscles from c.620A>T homozygotes. (**a**) XHMM plot of chr9: 36,000,000–38,000,000. No large deletions or insertions were found in *GNE* in P1–3 or H1. (**b**) Sashimi plots of *GNE* transcripts in skeletal muscle showing that there were no structural variants in P1–P3.

No large sequence deletions or insertions were detected in *GNE* in c.620A>T homozygotes (P1–3, H1) (Fig. 2a). As mentioned above, haplotype-specific variants were identified in intronic or intergenic regions around *GNE*, but we did not interpret their pathogenic significance. Therefore, we analyzed the structure and expression rate of *GNE* transcripts, and no variants or structural changes were observed in *GNE* transcripts in skeletal muscle from P1–P3 (Fig. 2b) or in fibroblasts from H1.

**Figure 3:**
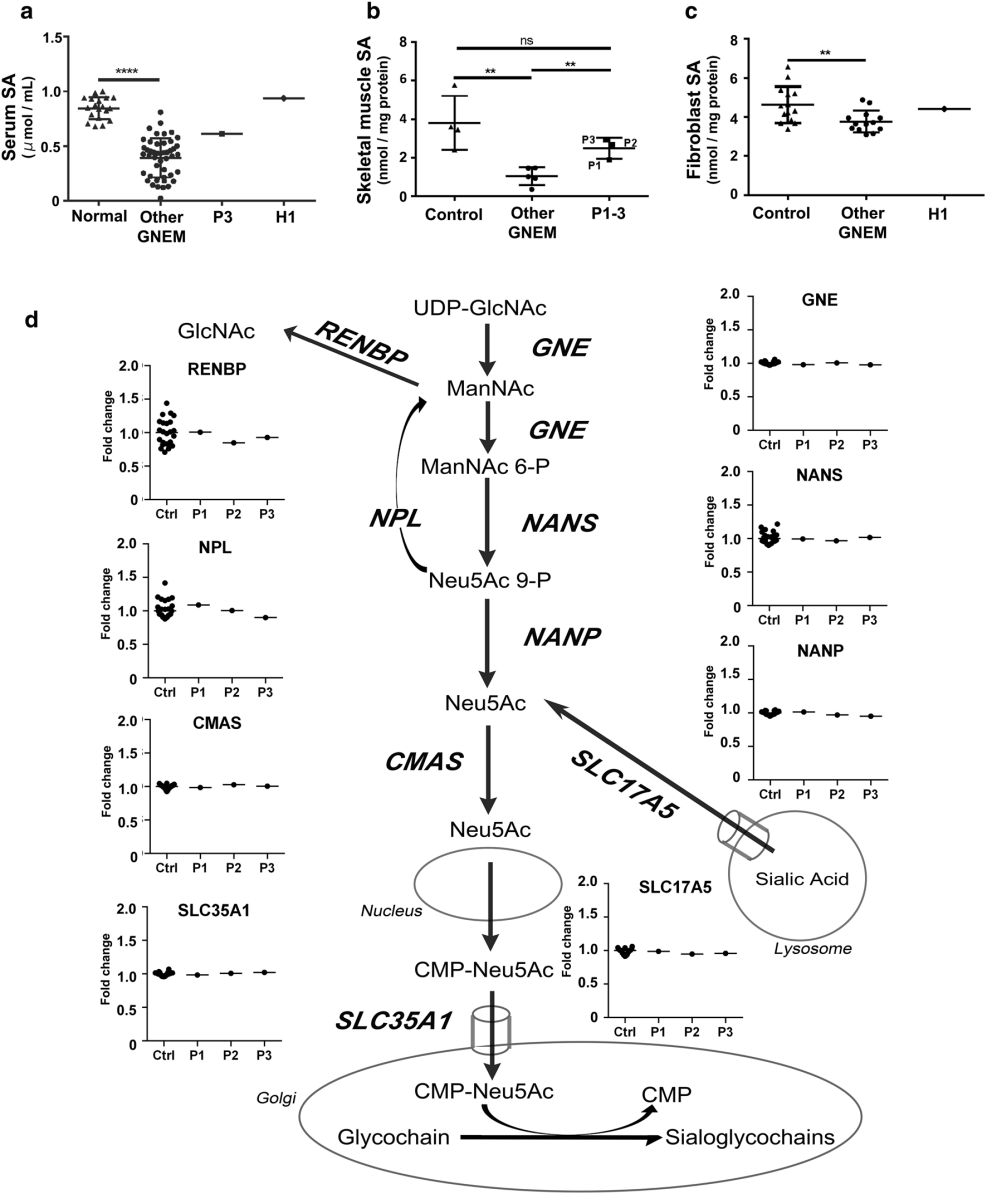
SA level and skeletal muscle transcript expression levels of genes involved in the SA metabolic pathway in c.620A>T homozygotes. (**a**) Serum SA concentrations in patients with GNE myopathy carrying other than c.620A>T homozygous mutation (n = 47) were significantly lower than those in healthy individuals (n = 19) (p < 0.0001). Serum SA concentration of P3 was in the upper range of that in patients with GNE myopathy carrying other than c.620A>T homozygous mutation, while that of H1 was in the range of healthy controls. (**b**) SA levels in skeletal muscles from patients with GNE myopathy carrying other than c.620A>T homozygous mutation (n = 6) were significantly lower than those of the disease controls (n = 4) (p = 0.0041), who were suspected to have muscle disease but whose muscle biopsy specimens exhibited no changes, while that of c.620A>T homozygous patients (n = 3, P1–3) was higher than those of other GNE myopathy patients (p = 0.0066) and lower than those of controls (p = 0.19) although the difference was not significant. (**c**) SA concentrations in fibroblasts from patients with GNE myopathy carrying other than c.620A>T homozygous mutation (n = 13) were significantly lower than those of disease controls (n = 14) (p = 0.0087), who were diagnosed as other muscle diseases. SA concentration in fibroblasts from H1 was in the control range. *p < 0.05, **p < 0.01, ***p < 0.001, ****p < 0.0001. (**d**) Transcript expression levels of genes associated with SA metabolism, including *GNE*, *CMAS*, *NANS*, *NANP*, *NPL*, *RENBP*, *SLC17A5*, and *SLC35A1*, were normal in skeletal muscle of three c.620A>T homozygotes (P1–3). Fold-change indicates the ratio of counts per million divided by the median value in controls.

Hyposialylation in muscle tissues, due to reduced SA production, is considered to be associated with muscle weakness in GNE myopathy. Therefore, we measured SA in serum samples from P3 and H1, and found that they were lower in P3 than those in healthy controls and similar to those in patients with GNE myopathy carrying other than c.620A>T homozygous mutation, while in H1 they were higher than those in patients with GNE myopathy, including P3 (Fig. 3a). SA levels in muscles from P1–3 were significantly higher than those in patients with GNE myopathy carrying other than c.620A>T homozygous mutation, while there was no significant difference from controls (Fig. 3b). SA levels in fibroblasts were significantly lower in patients with GNE myopathy carrying other than c.620A>T homozygous mutation than those in controls, while those of H1 were in the control range (Fig. 3c). Thus, SA levels correlated well with phenotypes.

We also analyzed genomic sequences of P1–3 and H1, and RNA expression of eight genes (*CMAS*, *GNE*, *NANP*, *NANS*, *NPL*, *RENBP*, *SLC17A5*, and *SLC35A1*), other than *GNE*, that are associated with SA biosynthesis and metabolism, in skeletal muscles of P1–3. No structural variation was observed, and mRNA expression levels of all eight genes were within normal ranges (Fig. 3d). Rare nonsynonymous exon and splicing variants found in those genes are listed in Supplementary Table 2.

### GNE aspartic acid 176 is not in the protein catalytic site, but may contribute to substrate transfer or oligomer formation

**Figure 4:**
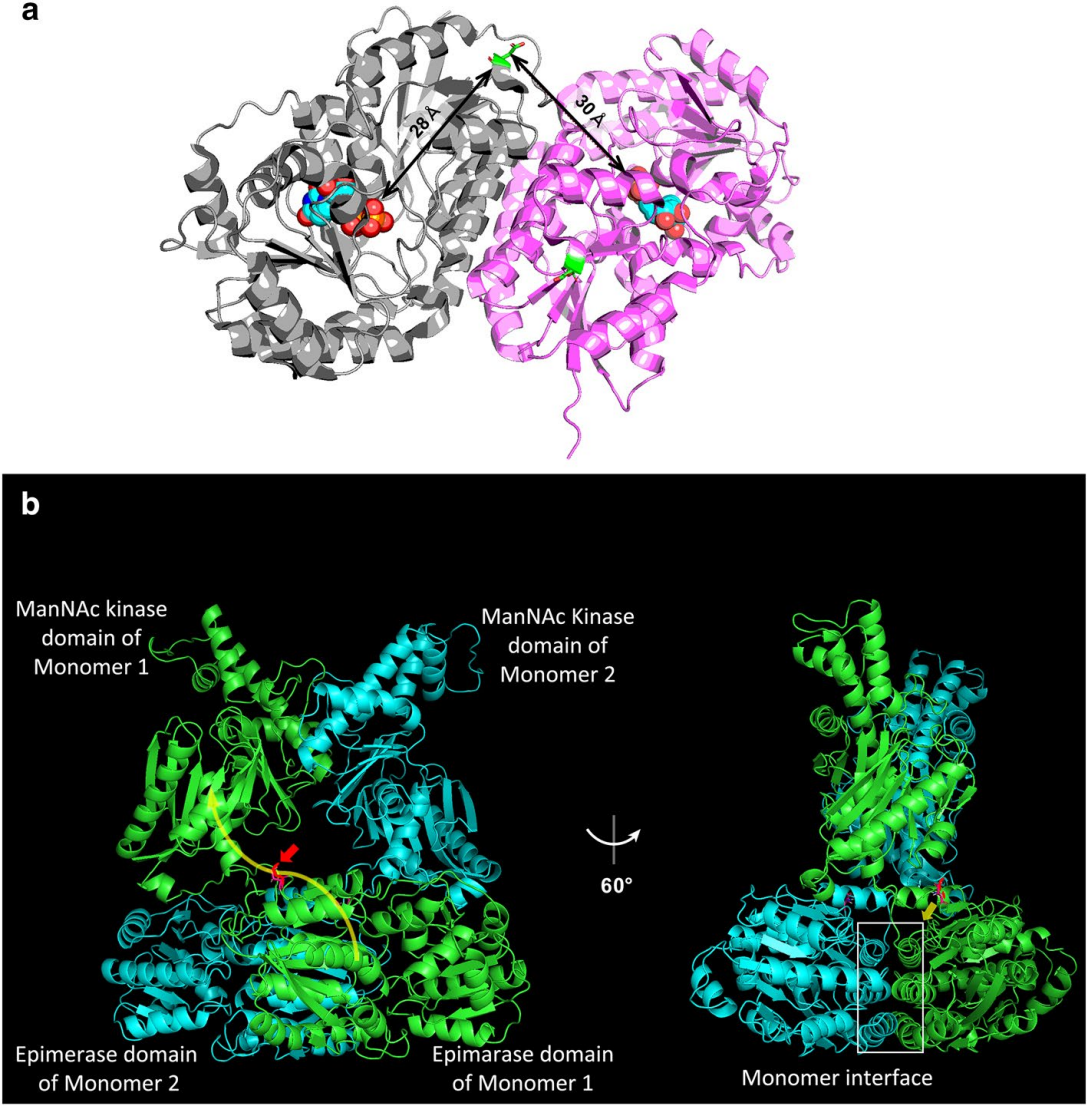
Location of aspartic acid 176 in a GNE protein structural model. (**a**) Structure of the epimerase domain of GNE according to Protein Data Bank (4ZHT). Aspartic acid 176 is located outside of the catalytic site (at 28 Å within the same domain and 30 Å from the other monomer). (**b**) Full-length GNE protein structure predicted using AlphaFold2. Left: Illustration of a GNE dimer consisting of monomer 1 (light blue) and monomer 2 (light green). Aspartic acid 176 (red) is located on the surface of the epimerase domain, which may be involved in intramolecular ManNAc transfer (yellow arrow in monomer 1). Side view of the GNE complex rotated by 60°. Aspartic acid 176 is located near the dimer-dimer interface but does not appear to be directly involved in the interaction between the two monomers.

The c.620A>T variant encodes a missense p.D207V variant in the longest GNE protein isoform. We explored the possible roles of this amino acid substitution on GNE enzyme structure by mapping aspartic acid residue 176 of the short GNE isoform (corresponding to aspartic acid 207 in the longest GNE isoform) on the crystal structure of the epimerase domain, and found that it is not located within, and is distant from (28 Å and 30 Å, intra- and intermonomer distances, respectively), the catalytic sites (Fig. 4a). Structural modeling of the full-length GNE molecule epimerase and kinase domains using AlphaFold2 revealed that GNE molecules form a stable dimer. Aspartic acid 176 is located on the surface of the epimerase domain, as previously reported^12^, and near to the interface between the epimerase domain of each monomer, but does not appear to be directly involved in their interaction (Fig. 4b), and only occasionally appears to form hydrogen bonds with the other monomer. The generated structure also suggested the possibility that this aspartic acid residue functions in ManNAc transfer from the epimerase domain to the kinase domain within GNE molecules (Fig. 4b). Thus, substitution of aspartic acid to valine at this site could account for failure of ManNAc transfer by altering the polarity or charge of the amino acid side chain.

## Discussion

In this study, we focused on the *GNE* variant, c.620A>T, which is the second most frequent in the Japanese general population. The majority of c.620A>T homozygotes are not diagnosed with GNE myopathy, while most compound heterozygotes that carry this variant are assumed to develop myopathy. There are two possible reasons why homozygotes are not identified as patients: either homozygosity mostly results in embryonic lethality, or the majority of homozygous individuals do not develop myopathy throughout their life. Embryonic lethality is unlikely, due to the identification of a healthy homozygous individual and the fact that the three identified patients also showed milder phenotypes and less SA reduction in skeletal muscle. Moreover, the age of onset was relatively high in compound heterozygotes carrying c.620A>T, suggesting that most individuals homozygous for this variant have an extremely mild phenotype or do not develop myopathy.

To evaluate whether c.620A>T originated from a common ancestor, we performed IBD mapping in four homozygotes and found two distinct haplotypes derived from different ancestors: Haplotype α, shared by P1 and P2, and Haplotype β, shared by P3 and H1. The haplotypes did not correlate with clinical characteristics because: (1) homozygotes for both Haplotypes α and β included symptomatic patients, demonstrating that homozygotes of either haplotype can develop disease; (2) frequencies of both haplotypes were maintained in both compound heterozygous patients with GNE myopathy and healthy heterozygous carriers; and (3) there was no significant difference in age at onset between compound heterozygous patients carrying Haplotypes α or β, indicating that patients with both haplotypes have the same phenotype.

The pathogenicity of c.620A>T is of interest. There is good evidence that this variant is pathogenic including: (1) the number of patients, since 179 compound heterozygous patients carrying this variant and another variant were identified; (2) the in vitro epimerase activity of the GNE short isoform with a corresponding mutation is approximately 18% of that of wild-type GNE^13^; and (3) a transgenic mouse model expressing human GNE with a corresponding mutation recapitulated GNE myopathy phenotypes^14^. However, based on the current study, the pathogenicity of c.620A>T cannot be simply interpreted, as the variant has the unusual feature of behaving as pathogenic in a compound heterozygous state, while it mostly does not when homozygous. We hypothesized that the three homozygous patients (P1–3) exceptionally have additional pathogenic variants, which cause hyposialylation in serum, skeletal muscles, and fibroblasts, since most individuals homozygous for c.620A>T do not develop hyposialylation. Nonetheless, no additional variants and no differences in expression of all genes involved in SA biosynthesis, including *GNE*, were detected in the three patients homozygous for c.620A>T. From our analysis, we conclude that the three c.620A>T homozygous patients are unlikely to carry additional pathogenic genetic abnormalities in the de novo SA biosynthesis pathway.

**Figure 5:**
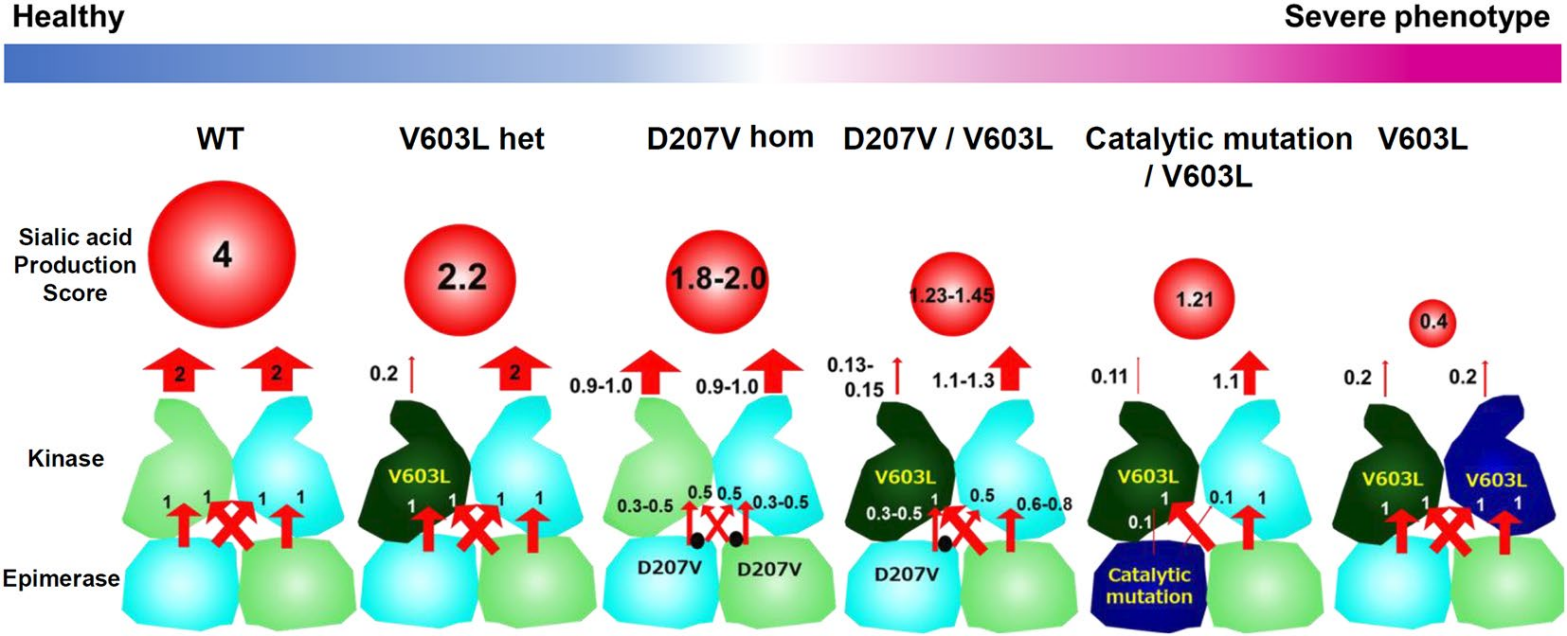
Model of ManNAc 6-phosphate production by mutated GNE. ManNAc 6-phosphate production ability was predicted as the sum of production route scores, which were calculated by multiplying the UDP-GlcNAc 2-epimerase reaction, ManNAc transfer, and ManNAc kinase reactions in the GNE dimer. Enzymatic reactions mediated by UDP-GlcNAc 2-epimerase and ManNAc kinase, and intramolecular and intermonomer ManNAc transfer in normal GNE dimer: Score 1. In GNE molecule with D207V, intramolecular ManNAc transfer may be affected: Score 0.5. Intermonomer transfer in GNE dimer with D207V is variable, due to failure of oligomerization: Score 0.3–0.5. In mutant GNE with catalytic mutations or V603L, both enzymatic reactions are affected: Score 0.1 and 0.2, respectively.

The essential cause of muscle weakness in GNE myopathy has been wildly accepted to be hyposialylation based on model mice study^4,5^ and low suppression of reactive oxygen species (ROS) production due to hyposialylation has been also reported as one of the disease mechanisms^15^. The mild reduction in sialylation might preserve partial ability of scavenging ROS leading to a mild form of the disease. It may be attributable to the location and function of the substituted residue in the GNE protein. D176V in the short GNE isoform, a missense variant corresponding to D207V in the long GNE isoform and encoded by c.620A>T, is unlikely to induce a drastic change in overall GNE structure, according to in silico simulation analysis. The location of the aspartic acid at position 176 is on the protein surface, far from catalytic sites, which also suggests that it is unlikely to directly influence enzymatic activity. The structural model indicated that the residue is located in a tract involved in intramolecular transfer of ManNAc, the enzymatic product of the first epimerase reaction, from the epimerase domain to the kinase domain. The replacement of a hydrophilic polar amino acid (aspartic acid) with a hydrophobic nonpolar amino acid (valine) will decrease the affinity for ManNAc, thereby reducing transfer efficiency. In addition, this residue is located behind the monomer–monomer interface and could contribute indirectly to interaction between the epimerase domains of each monomer. Interestingly, we have previously reported in vitro analysis showing that oligomerization of GNE with the D176V mutation was partial, but not complete^13^, consistent with the findings of structural analysis. Failure of oligomerization is likely to cause a defect in ManNAc transfer between monomers (epimerase domain of monomer 1 to kinase domain of monomer 2 or the reciprocal exchange). Defects in ManNAc transfer by both intramolecular and intermonomer routes will lead to a decrease in ManNAc 6-phosphate production via the two enzymatic reactions mediated by GNE. We illustrate our hypothetical model for c.620A>T variant pathogenicity in Fig. 5. Patients homozygous for GNE D207V will have variable ManNAc 6-phosphate production, depending on its oligomer status, while compound heterozygotes for this variant and a catalytic site variant are predicted to produce less ManNAc 6-phosphate. Furthermore, as this residue is located on the surface of the protein, the amino acid substitution may alter protein susceptibility to post-translational modifications and/or the cytosolic environment, which are also suggested to modify GNE enzymatic activity. This hypothetical model well correlates with the sialic acid production level and phenotype of each variant, though still speculative as there is an experimental limitation to prove the degree of ManNAc transfer. Further experimental studies are needed to elucidate the precise effects of this amino acid replacement on GNE oligomer functions, which could be a therapeutic target.

In patients with GNE myopathy in India, the c.2179G>A variant is reported to have similar characteristics to c.620A>T in Japan; of 54 patients genetically diagnosed with GNE myopathy, 38 were compound heterozygotes carrying c.2179G>A, along with only one homozygote^16^, and the phenotypes of patients carrying this variant are reported to be mild, with relatively later onset and wheelchair use, in that cohort^17^. These data suggest that c.620A>T is not the only variant leading to discrepancies between disease development in homozygotes and compound heterozygotes. Studies of c.620A>T, together with those of c.2179G>A, may provide clues to aid understanding of these phenomena.

The findings of this study provide new insights into the pathogenicity of GNE variants and the pathomechanisms underlying GNE myopathy, and have important implications for the development of new therapeutic options. Patients carrying variants outside of the catalytic sites may be rescued. In particular, in those carrying c.620A>T, small molecules that can enhance oligomerization of the GNE protein are a potentially promising approach. Identification and analysis of affected and heathy c.620A>T homozygotes will be helpful in achieving these aims.

## Methods

### Subjects

Japanese patients with GNE myopathy (n = 365) until December 2021 were genetically confirmed with biallelic pathogenic variants, including three c.620A>T homozygotes (P1–P3). Genomic analysis of samples from unaffected families was also conducted and identified a healthy individual homozygous for c.620A>T (H1), who is the father of a patient with GNE myopathy carrying *GNE* c.620A>T and c.1085T>C. Informed consent and approval by the NCNP Ethical Review Board were obtained for collection of clinical data and extraction of DNA for analysis.

### Clinical information

A national registry for neuromuscular diseases in Japan (Registry of Muscular Dystrophy; Remudy, http://www.remudy.jp/) was developed in 2009 in collaboration with Translational Research in Europe-Assessment and Treatment of Neuromuscular Disease^18^. The registry was expanded to include patients with GNE myopathy in 2012^19^. Participants were genetically confirmed with biallelic pathogenic variants in *GNE*, or those with monoallelic pathogenic variants were registered when rimmed vacuoles were observed in muscle specimens. A total of 212 Japanese patients with GNE myopathy, registered until March 2021, were included. Detailed clinical information for P1–3 and H1 is provided in Supplementary information 1.

### Estimation of the number of homozygotes in the Japanese general population

The national population in Japan was based on current population estimates, as of November 1, 2021, published by the Statistics Bureau of Japan (https://www.stat.go.jp/english/data/kokusei/2020/summary.html). Allele frequencies of c.620A>T and c.1807G>C in the Japanese population were based on genotyping of 1890 participants in the Nagahama cohort study^20^. We estimated the number of homozygotes in the Japanese population using following equation: [Estimated number of homozygotes] = [Estimated Japanese population/Average age of onset of GNE myopathy in Japanese patients in Remudy] × [variant allele frequency]^2^.

### Variant annotation

Variation nomenclature used in this manuscript is based on the longest transcript (GenBank: NM_001128227), following the guidelines of the Human Genome Variation Society (HGVS; http://www.hgvs.org/content/guidelines). Human *GNE* comprises 13 exons, although no splice variants include all exons; therefore, the nomenclature and exon numbering suggested in a previous article was adopted^21^. Deleterious single nucleotide variants were scored using Combined Annotation Dependent Deletion (https://cadd.gs.washington.edu/). Variant allele frequencies were compared with those in the public database at gnomAD (https://gnomad.broadinstitute.org/). The human reference genome annotated with University of California Santa Cruz gene annotation (hg19) served as the reference sequence.

### Direct sequencing and targeted next-generation sequencing

Direct sequencing of *GNE* and targeted next-generation sequencing were performed using genomic DNA (50–100 ng) extracted from peripheral blood leukocytes or frozen skeletal muscle tissue (10 μm/slice × 100 slices) collected for diagnosis to 365 Japanese GNE myopathy patients and 9 unaffected families. Direct sequencing was performed by amplifying each exon and flanking regions of *GNE* by PCR using primer sets described previously^22^. Multiplex primer pools were designed using Ion Ampliseq Design software (Thermo Fisher Scientific). The custom *GNE* panel covered all exons (99.3% coverage) and flanking regions (5 bp from exon–intron boundaries). Target regions were enriched using an Ion AmpliSeq Library Kit 2.0 (Thermo Fisher Scientific) and sequenced on an Ion PGM (Thermo Fisher Scientific) using Ion 318 Chip (Thermo Fisher Scientific), according to the manufacturer’s protocol. Copy number variation (CNV) analysis was conducted according to Zhu et al.^23^.

### Genome sequencing

Genome sequencing was conducted using 10 × Genomics linked-read sequencing (10 × Genomics) or the BGISEQ-500 high-throughput genome sequencing platform. CNVs were assessed by 10 × Genomics linked-read sequencing or eXome-Hidden Markov Model (XHMM)^24^, using sequence data from the BGISEQ-500 high-throughput genome sequencing platform. Nonparametric linkage analysis was conducted, which enabled IBD mapping based on the “SNP streak” approach, to assess the homogeneity of adjacent SNP genotypes in the ancestral haplotype, as described previously^25,26^. Genome sequencing data from individuals homozygous for c.620A>T from the Biobank Japan Database [https://searchweb.svc.biobankjp.org/ (in Japanese)] and data from the Nagahama cohort study were used for haplotype analysis of patients with compound heterozygosity and heterozygotes, and to distinguish the two founder haplotypes carrying c.620A>T by analyzing SNP patterns of four common variants. Rare nonsynonymous exonic and splicing variants, with frequencies < 0.001 in gnomAD total or East Asian populations, in genes associated with SA biosynthesis (*CMAS*, *NANP*, *NANS*, *NPL*, *RENBP*, *SLC17A5*, and *SLC35A1*) were also explored.

### Cell culture

Primary fibroblasts isolated from each of H1, GNE myopathy patients carrying other than c.620A>T homozygous mutation (n = 13), and disease controls (n = 14) were cultured with 10% fetal bovine serum in DMEM/Ham’s F-12 medium (Sigma) in a humidified chamber with 5% CO_2_ at 37 °C in a 10 cm dish. Culture medium was replaced with serum-free DMEM/Ham’s F-12 for 96 h before harvest. Cells were harvested by trypsin digestion, and recovered cells were washed twice with PBS. Collected cells were divided into two tubes: one for SA measurement and the other for total protein assay.

### RNA-seq

Total RNA (20–50 ng) was extracted from frozen skeletal muscle tissue (10 μm/slice × 100 slices) of P1–P3 and 24 disease controls using a PureLink RNA Mini Kit (Thermo Fisher Scientific), purified using PureLink DNase Treatment (Thermo Fisher Scientific), and cDNA (20–50 ng) synthesized with SuperScript IV Reverse Transcriptase (Thermo Fisher Scientific) using Oligo(dT) 15 Primer (Promega). cDNA analysis of fibroblast-derived RNA (20–50 ng) of H1 was also conducted, using primers designed to amplify the full-length *GNE* cDNA (Supplementary Table 1) and the synthesized cDNA amplified using PCR Master Mix (Promega). Products were purified from agarose gel using a QIAquick Gel Extraction Kit (QIAGEN) and sequenced. Muscle-derived RNA (1 µg) was sequenced on the Illumina Hiseq Platform (Illumina), generating approximately 13.07 Gb of sequence per sample; clean reads were mapped to the reference genome using HISAT^27^ and Bowtie^28^, transcripts were reconstructed using String Tie^29^, and gene expression levels were calculated with RSEM^30^. Sashimi plots generated using the mixture-of-isoforms model were used to map bam files and visualize aberrant splicing events^31^. Quantitative visualizations of patient RNA-seq data were compared with those of controls and publicly available information about transcript expression levels in each tissue (https://gtexportal.org/home/transcriptPage).

### Measurement of SA concentration and total protein content

Total SA was measured in plasma isolated from whole blood by centrifugation (10 min at 2000×*g*), frozen skeletal muscle specimens, and cultured fibroblasts, as described previously^32,33^. Serum SA concentrations were measured in c.620A>T homozygotes (P1-3, H1), patients with GNE myopathy carrying other than c.620A>T homozygous mutation (n = 47), and healthy individuals (n = 19). SA levels in skeletal muscles were measured in c.620A>T homozygous patients(P1–3), patients with GNE myopathy carrying other than c.620A>T homozygous mutation (n = 6) and the disease controls (n = 4). SA concentrations in fibroblasts are measured in H1, patients with GNE myopathy carrying other than c.620A>T homozygous mutation (n = 13), and disease controls (n = 14). Protein was extracted in SDS buffer (2% SDS, 0.0625 M Tris–HCl, pH 6.8) and quantified using a Bio-Rad Protein Assay (Bio-Rad Laboratories, Herecules), following the manufacturer’s instructions. SA concentrations were compared to those of the healthy controls or the disease controls using Student’s t-test (two-tailed). P < 0.05 was considered statistically significant.

### Structural modeling

Crystal structures of the UDP-2-epimerase domain of the GNE short isoform were obtained from Protein Data Bank (https://www.rcsb.org/; UDP-2-epimerase domain, 4ZHT). Distances between asparagine 176 and catalytic sites were measured using PyMOL software. The full-length structure of the GNE short isoform and its dimerization model were constructed using AlphaFold2 software (https://github.com/YoshitakaMo/localcolabfold)^34–38^.

### Statistical analyses

We performed all statistical analyses using Graph Pad Prism 5.0 (GraphPad Software, Sann Diego, CA). We used the Wilcoxon matched-pairs signed rank test to compare two unrelated groups and the x^2^ test for trend to estimate correlation analysis in a scatter plot, which corresponds to 95% confidence intervals. P value < 0.05 was set as the threshold for statistical significance.

### Ethics declarations

This study involved human participants and was approved by the ethical committee of the National Center of Neurology and Psychiatry. The ethics approval ID is XXXX-116. Participants gave informed consent to participate in the study before taking part.

### Consent to participate

Informed consent was obtained from individual participants included in the study.

## Supplementary Information

Supplementary Information 1.

Supplementary Tables.

## Data Availability

The datasets generated and/or analyzed during the current study are available in the Dryad, Dataset, https://datadryad.org/stash/share/bOVGL-lPa-fu029DfhC1f2-6GO-_Vlov1VXAoqaeprs.
