# Supplementary material for: Multidimensional analyses of the pathomechanism caused by the non-catalytic GNE variant, c.620A>T, in patients with GNE myopathy
Source: Sci Rep. 2022 Dec 16;12:21806. doi: 10.1038/s41598-022-26419-0 (PMC9758176; doi:10.1038/s41598-022-26419-0)
Supplement: Supplementary file 1 — Supplementary Information 1. [file 41598_2022_26419_MOESM1_ESM.docx]

**Supplementary information1：Clinical feature of three p.D207V homozygous patients (P1-3)**

Patient1 (P1) is a Japanese male who noticed that he could not throw a ball far at the age of 37 years and lost ability to run 3 years later. At the age of 44 years, he visited a hospital with difficulty in climbing stairs. Serum creatine kinase (CK) level was 580 IU/l. Manual Muscle Testing (MMT) grades were 4-/3 for the *tibialis anterior*, 2-/2- for *iliopsoas*, and 5/5 for *quadriceps femoris* muscles. Patient2 (P2), a Japanese male, recognized frequent stumbling and difficulty in carrying heavy boxes at the age of 34 years. Six years later, he admitted hospital with difficulty in walking. Serum CK level was 474 IU/l. MMT grades were 3-/1 for the *tibialis anterior*, 3+/3 for *iliopsoas*, and 4/3+ for *quadriceps femoris* muscles. Patient3 (P3), a Japanese male, recognized frequent stumbling at the age of 60 years and visited hospital 5 years later. CK level was 401 IU/l. MMT grades were 3/3 for the *tibialis anterior*, 4/4 for *iliopsoas*, and 4/4 for *quadriceps femoris* muscles. Muscle biopsy of all three patients showed variable fiber size and degenerated atrophic myofibers with rimmed vacuoles. Neither of the patients had family history of muscle weakness nor consanguineous marriage.
