# Supplementary material for: Multidimensional analyses of the pathomechanism caused by the non-catalytic GNE variant, c.620A>T, in patients with GNE myopathy
Source: Sci Rep. 2022 Dec 16;12:21806. doi: 10.1038/s41598-022-26419-0 (PMC9758176; doi:10.1038/s41598-022-26419-0)
Supplement: Supplementary file 2 — Supplementary Tables. [file 41598_2022_26419_MOESM2_ESM.docx]

| **GNE specific primer for cDNA synthesis** | | | | | |
| --- | --- | --- | --- | --- | --- |
| Exon | Sequence 5’>3’ | | | | |
| 13 | GGATGACCGTTTCTTAACAATCA | | | | |
| **GNE specific primer for cDNA amplification** | | | | | |
|  | | Forward | | Reverse | |
|  | | Exon | Sequence 5’>3’ | Exon | Sequence 5’>3’ |
| Fibroblast cDNA | | 1-2 | GCGTCTGGAACTCTATTTTAAG | 13 | GGATGACCGTTTCTTAACAATCA |
| Skeletal muscle cDNA | | 2 | GAGTGGGGACAAGGTCGAG | 4 | ATGGAGACAGGTTTGATGCCC |
|  |  | 3 | CTGCGGGTTTGTGTTGCTAC | 5 | TGTTGCACTACAGCACCCTGTG |
|  |  | 4 | GTGAAGCTGCCAGATGTCCT | 10 | CAGGTGGCCGTGTAAATCCT |
|  |  | 7 | TGCTGACACCCAAGACAAAA | 13 | GGATGACCGTTTCTTAACAATCA |

**Supplementary Table 1 Primers for cDNA analysis**

**Supplementary Table 2 Rare variants in genes associated with sialic acid metabolism**

| Gene | Chr | NM | Ex |  | P1 | P2 | P3 | H1 | HGMD_  phenotype | HGMD  inheritance |
| --- | --- | --- | --- | --- | --- | --- | --- | --- | --- | --- |
| NANS | 9 | NM_018946.3 | 6 | c.977_978insCAT  (p.D326delinsDI) | . | . | . | het | Developmental delay & skeletal dysplasia, infantile-onset | AR |
| NPL | 1 | NM_030769.2 | 9 | c.606+8->C | . | . | . | het | unreported | |

Chr: chromosome, Ex: exon, .: no variant (reference sequence), het: heterozygous variant
